# Coherent DOA Estimation of Multi-Beam Frequency Beam-Scanning LWAs Based on Maximum Likelihood Algorithm

**DOI:** 10.3390/s25123791

**Published:** 2025-06-17

**Authors:** Yifan Yang, Rihui Zeng, Qingqing Zhu, Weijin Fang, Biyun Ma, Yide Wang

**Affiliations:** 1 China Mobile Group Design Institute Co., Ltd. Guangdong Branch, Guangzhou 510623, China; yangyifan@cmdi.chinamobile.com (Y.Y.); zengrihui@cmdi.chinamobile.com (R.Z.); fangweijin@cmdi.chinamobile.com (W.F.); 2 School of Electronic and Information Engineering, South China University of Technology, Guangzhou 510640, China; eeqqzhu@mail.scut.edu.cn; 3 Institut d’Electronique et des Technologies du Numérique (IETR), Centre National de la Recherche Scientifique (CNRS), Unité Mixte de Recherche (UMR) 6164, Nantes University, 44306 Nantes, France; yide.wang@univ-nantes.fr

**Keywords:** direction-of-arrival estimation, frequency scanning leaky-wave antenna, multi-beam scanning

## Abstract

Multi-Beam frequency scanning leaky-wave antennas (FBS-LWAs) offer a viable solution for hardware miniaturization in direction-of-arrival (DOA) estimation systems. However, the presence of multiple spatial harmonics results in responses in multiple directions for a given incident source, introducing estimation ambiguity and significantly challenging accurate DOA estimation. Moreover, due to the nonlinear frequency response of the FBS-LWA, its response matrix does not satisfy the Vandermonde structure, which renders common rank-recovery techniques ineffective for processing coherent signals. As a result, the DOA estimation of coherent sources using multi-beam FBS-LWAs remains an open and challenging problem. To address this issue, this paper proposes a novel DOA estimation method for coherent signals based on multi-beam frequency scanning leaky-wave antennas. First, the received signals are transformed into the frequency domain via fast Fourier transform (FFT) to construct the signal data matrix from which the covariance matrix is computed.Then, conventional beamforming (CBF) is employed to obtain an initial estimate of the angle set, which will be further refined by a smaller grid to form a candidate angle set. Finally, a maximum likelihood algorithm based on the stochastic principle (Sto-ML) is used to suppress the interference of the parasitic directions and select the final DOA estimates from the candidate angle set. Simulation results show that the proposed method effectively mitigates the impact of parasitic directions and achieves an accurate DOA estimation of multiple coherent sources, even under both low and medium-to-high signal-to-noise ratio (SNR) conditions.

## 1. Introduction

Direction-of-arrival (DOA) refers to the direction angle at which signals reach the reference element of an antenna array. As a fundamental concept in spatial spectrum estimation theory, DOA estimation has become a critical research area in array signal processing, with extensive applications in radar, sonar, wireless communications, medical imaging, and other domains [1,2,3]. Traditional DOA estimation systems consist of antenna arrays, analog-to-digital conversion (ADC) modules, and signal processing units, relying on a large number of radio frequency (RF) channels. While more RF channels enhance estimation accuracy by reducing received signal correlation, they simultaneously increase system complexity and manufacturing costs. Recent advances in self-powered reconfigurable intelligent surfaces (RISs) [4] also illustrate the increasing complexity and flexibility in array design. Consequently, simplifying the hardware implementation of DOA estimation devices remains a key research priority [5,6].

Leaky-wave antennas employ a guiding structure that supports wave propagation along the waveguide structure. Unlike phased arrays, LWAs achieve beam scanning without complex feeding networks or active components such as phase shifters, thereby simplifying structural design and reducing manufacturing complexity and cost. This technology has found extensive applications in satellite positioning, radar detection, and other fields [7,8,9]. Since its invention in the 1940s, LWA configurations have undergone continuous evolution, with microstrip lines, coplanar waveguides, and substrate-integrated waveguides emerging in succession [10,11,12]. Post-2014 advancements, driven by composite right/left-handed (CRLH) metamaterials and dielectric materials like liquid crystals, have propelled LWA development towards miniaturization, wide-angle scanning capabilities, and customizable form factors [13,14,15]. Among these innovations, frequency beam-scanning LWAs (FBS-LWAs) steer their beam direction by varying the operating frequency, offering a simpler design compared to their electronically controlled counterparts. Moreover, multi-beam FBS-LWAs exploit multiple Floquet harmonics to scan the entire field of view (FoV) using a lower frequency bandwidth compared to their single-beam counterparts, making them a promising candidate for the miniaturization of DOA estimation systems [16]. Similarly, the concept of multi-beam beamforming has been extended to time-modulated arrays for automotive radar applications, further demonstrating the versatility of multi-beam architectures in practical scenarios [17].

In the field of DOA estimation for single-beam FBS-LWAs, the estimation of uncorrelated sources has been effectively addressed using power-based and subspace-based methods, while the related work for coherent sources faces the following issues:

(1) Classical DOA estimation algorithms such as the MUSIC algorithm (multiple signal classification) and the ESPRIT algorithm (estimation of signal parameters via the rotational invariance technique) are not suitable for coherent sources [18,19]. To solve the problem of source correlation, preprocessing using spatial smoothing algorithms such as MSSP (modified spatial smoothing preprocessing) or FBSS (forward-backward spatial smoothing) is required. However, these methods come at the cost of sacrificing the aperture, which in turn reduces the estimation accuracy of the DOA algorithm [20,21]. In addition, spatial smoothing algorithms require the response matrix to satisfy the Vandermonde structure, but the FBS-LWA does not meet this requirement.

(2) DOA algorithms such as MODE (method of direction estimation) and PUMA (principal singular vector utilization modal analysis) are capable of resolving coherent signals and achieving high resolution [22,23]. However, these methods rely on the assumption that the antenna response matrix possesses a Vandermonde structure, which is not satisfied by FBS-LWAs.

To enable the DOA estimation of coherent sources using single-beam FBS-LWAs, [24] first employs the conventional beamforming (CBF) algorithm to obtain an initial angle estimate. This estimate is then used to construct an interpolation matrix that transforms the antenna response into a Vandermonde structure. Subsequently, the modified enhanced PUMA (Mod-EPUMA) algorithm, combined with the FBSS technique, is applied to achieve the DOA estimation of coherent sources. It is worth noting that the accuracy of this interpolation-based method heavily depends on the precision of the initial angle estimates.

On the other hand, multi-beam operation poses an additional challenge to DOA estimation. Due to the presence of multiple spatial harmonics, the spectrum obtained by the CBF algorithm exhibits multiple peaks, including the true DOAs and several parasitic directions. The presence of the latter introduces the parasite interference to DOA estimation, which degrades the performance of conventional DOA estimation methods and makes the interpolation-based method unavailable for multi-beam FBS-LWAs.

To address the above issues, [25] proposes a low-complexity DOA estimation method for FBS-LWAs based on the concept of group fusion. The wideband signals received by multi-beam FBS-LWAs are grouped by frequency samples to partially recover the rank of the covariance matrix. After constructing the covariance matrices and array manifold vectors corresponding to different groups, the MUSIC algorithm is then employed to perform DOA estimation. This scheme can achieve a DOA estimation of strongly correlated signals at a signal-to-noise ratio (SNR) of 25 dB. However, the dependence on a high SNR and strong coherence constrains its application in more general scenarios. Similarly, to recover the rank of the covariance matrix, [26] attempts to use the received data from two ports of multi-beam FBS-LWAs for a noise subspace average. However, this method has limited decoherence capability with a large number of sources and deteriorates when under complex parasitic interference scenarios. In conclusion, DOA estimation for coherent sources based on the multi-beam FBS-LWA received model remains an open and challenging problem.

The maximum likelihood (ML) [27] algorithm can achieve accurate DOA estimation without the requirement of a Vandermonde structured steering matrix [28], offering a feasible solution to DOA estimation based on multi-beam FBS-LWAs. Building on this insight, this paper proposes a novel DOA estimation method based on multi-beam FBS-LWAs. Specifically, the received signals are first transformed into the frequency domain via fast Fourier transform (FFT) to construct the signal data matrix, from which the covariance matrix is computed. An initial angle set is then obtained using the CBF algorithm, which is further refined using a smaller grid to generate a candidate angle set. Finally, an ML algorithm based on the stochastic principle (Sto-ML) is employed to suppress the interference caused by parasitic directions and to select the final DOA estimates from the candidate set. Simulation results demonstrate that the proposed method effectively mitigates the influence of parasitic responses and achieves accurate DOA estimation of multiple coherent sources, even under low-SNR regions and complex parasitic interference scenarios.

## 2. Multi-Beam FBS-LWA Received Model

### 2.1. LWA Model

**Figure 1:**
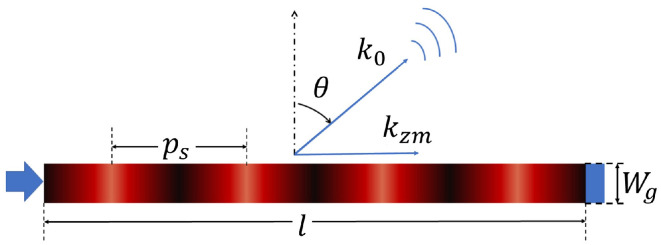
1-D periodic leaky-wave antenna (input port on the left, matched load on the right).

A multi-beam configuration of the FBS-LWA model [29] is considered in this paper, as shown in Figure 1, which utilizes a wideband signal to scan the whole FoV. Ignoring the cross-mode coupling, the response of the multi-beam FBS-LWA under a given angle θ and operating frequency $f_{n}$ can be modeled as

(1) $$a_{n}\left(\theta \right)=l\sum_{m=m_{\mathrm{back}}}^{m_{\mathrm{forw}}}\left\{e^{-\frac{j(k_{\mathrm{zm}}\left(n\right)-k_{0}\left(n\right)\sin\left(\theta \right))l}{2}}\mathrm{sinc}\left[\frac{(k_{zm}\left(n\right)-k_{0}\left(n\right)\sin\left(\theta \right))l}{2}\right]\right\}$$

where n=1,2,…,N represents the number of operating frequency samples. $f_{1}=f_{\min}$ is the minimum operating frequency, $f_{N}=f_{\max}$ is the maximum operating frequency, and $f_{n}$ is the nth operating frequency. l is the length of the antenna. m is the spatial harmonic order, and each harmonic contributes independently to the overall response. Therefore, Equation (1) models the linear superposition of responses from multiple spatial harmonics. $m_{\mathrm{back}}$ and $m_{\mathrm{forw}}$ are the harmonic orders radiated from the backward and forward endpoints, respectively, which are calculated by

(2) $$m_{back}=-\left\lfloor pf_{max}/c\left[1+\sqrt{\varepsilon_{r}}\sqrt{1-{\left(f_{c}/f_{max}\right)}^{2}}\right]\right\rfloor$$

(3) $$m_{forw}=\left\lfloor pf_{min}/c\left[1-\sqrt{\varepsilon_{r}}\sqrt{1-{\left(f_{c}/f_{min}\right)}^{2}}\right]\right\rfloor$$

where *p* is the spatial modulation period and *c* is the speed of light. $k_{\mathrm{zm}}\left(n\right)=\beta_{m}\left(n\right)-j\alpha_{n}k_{0}\left(n\right)$ is the longitudinal wavenumber inside the waveguide structure corresponding to the mth spatial harmonic order, where $\beta_{m}\left(n\right)=\beta_{0}\left(n\right)-2\pi m/p$ is the phase constant of the higher-order mode, $k_{0}\left(n\right)=2\pi f_{n}/c$ is the free-space wavenumber, and $\alpha_{n}$ is the attenuation constant accounting for radiative leakage and losses at frequency $f_{n}$. Then, $\beta_{0}\left(n\right)=k_{0}\left(n\right)\sqrt{\varepsilon_{r}}\sqrt{1-{\left(f_{c}/f_{n}\right)}^{2}}$ is the phase constant of the fundamental guided mode, with $\varepsilon_{r}$ being the relative permittivity of the dielectric material filling the waveguide, $f_{c}=\frac{c}{2W_{g}\sqrt{\varepsilon_{r}}}$ the cutoff frequency, and $W_{g}$ the waveguide width.

**Figure 2:**
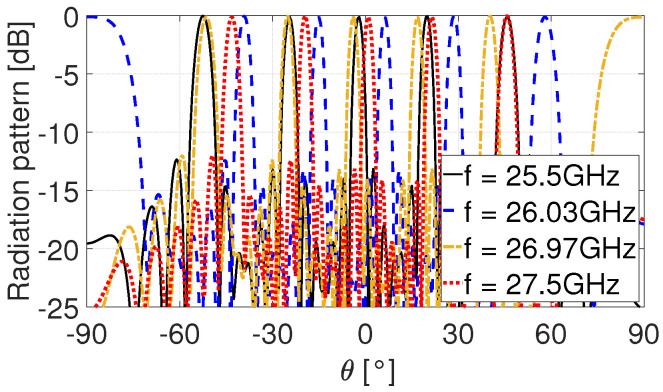
Normalized radiation pattern of multi-beam FBS-LWA obtained by (1).

Figure 2 illustrates the normalized radiation pattern of multi-beam FBS-LWAs, which is derived from (1). Owing to the simultaneous excitation of multiple harmonics, the antenna radiates multiple beams concurrently at a given operating frequency. As the frequency varies, the beam directions shift accordingly, allowing the multi-beam FBS-LWA to scan across the entire FoV with a reduced frequency bandwidth.

However, the multi-beam operation also introduces a critical issue: an incident source from a specific direction may excite responses in multiple directions due to the presence of multiple spatial harmonic components. As a result, the CBF spectrum of received signals exhibits several peaks, including the true DOA and other parasitic directions. These parasitic responses also persist in the spatial spectrum of the subspace-based algorithms, significantly degrading their estimation performance, which will be further analyzed and validated through simulations in Section 4.

### 2.2. Signal Model Received by Multi-Beam FBS-LWAs

Assuming that *K* sources impinge on the multi-beam FBS-LWA from directions $\theta_{k},\;k=1,2,\ldots ,K$, each source is captured by distinct spatial harmonics associated with different frequencies. The received electromagnetic waves are then linearly superimposed within the antenna’s structure. After ADC and other processing, an N−point FFT is applied to obtain the frequency domain data. After the compensation for transmission distortion, the signal received at the tth snapshot can be modeled as

(4) x(t)=As(t)+e(t)

where $x\left(t\right)\in C^{N\times 1}$ is the received signal vector, $A=\left[a\left(\theta_{1}\right),\ldots ,a\left(\theta_{K}\right)\right]\in C^{N\times K}$ is the response matrix of the multi-beam FBS-LWA, and $a\left(\theta_{k}\right)={\left[a_{1}\left(\theta_{k}\right),\ldots ,a_{N}\left(\theta_{k}\right)\right]}^{T}\in C^{N\times 1}$ is the response vector corresponding to the *k*th incident source. $s\left(t\right)\in C^{K\times 1}$ is the source vector and $e\left(t\right)\in C^{N\times 1}$ denotes the independent additive white Gaussian noise (AWGN).

The received signal that incorporates multiple snapshots is considered in this paper, which is expressed as follows:

(5) X=AS+E

where the received signal matrix is $X=[x(1),\ldots ,x(L)]\in C^{N\times L}$, the impinging signal matrix is $S=[s(1),\ldots ,s(L)]\in C^{K\times L}$, and the noise matrix is $E=[e(1),\ldots ,e(L)]\in C^{N\times L}$, where L denotes the number of snapshots. Then, the signal covariance matrix R can be calculated as

(6) $$R=\sum_{t=1}^{L}XX^{H}/L$$

where ${\left(•\right)}^{H}$ denotes the conjugate transpose of the vector.

## 3. Sto-ML-Based Coherent DOA Estimation Method for Multi-Beam FBS-LWAs

Given the high computational complexity of the Sto-ML algorithm, an initial DOA estimation is first conducted using the CBF algorithm, whose spectrum is expressed as

(7) $$P_{CBF}\left(\theta \right)=a{\left(\theta \right)}^{H}Ra\left(\theta \right)$$

where θ represents an arbitrary incoming angle within the FoV $\Theta_{FoV}$ and $\theta_{g}$ is the angular grid used to discretize the FoV.

As previously discussed, the spatial spectrum of CBF contains the true DOAs along with multiple parasitic peaks. Hence, the angles corresponding to the first KM peak points are selected to form the initial estimated angle set $\Theta_{ini}=\left[\theta_{1},\theta_{2},\ldots ,\theta_{P}\right]$, with P=KM and *M* as the number of antenna beams.

To enhance the estimation accuracy, a finer interval $\theta_{s}=\theta_{g}/2$ is employed around each initial estimate. This grid refinement yields a candidate angle set given by

(8) $$\Theta_{cand}=\left[\theta_{1}-\theta_{s},\theta_{1},\theta_{1}+\theta_{s},\ldots ,\theta_{P}-\theta_{s},\theta_{P},\theta_{P}+\theta_{s}\right]$$

For the sake of simplified expression, the above equation can be rewritten as

(9) $$\Theta_{cand}=\left[\vartheta_{1},\vartheta_{2},\ldots ,\vartheta_{P^{\prime }}\right]$$

where $P^{\prime }=3P$.

The next step of the proposed method is to identify the true DOAs from the candidate angle set $\Theta_{\mathrm{cand}}$. All possible combinations of *K* angles are formed from $\Theta_{cand}$, resulting in candidate groups $\left\{\Theta_{1},\Theta_{2},\ldots ,\Theta_{G}\right\}$, where the total number of groups *G* is given by

(10) G=P′K=P′!K!(P′−K)!

For a specific group $\Theta_{i}=\left[\vartheta_{i1},\vartheta_{i2},\ldots ,\vartheta_{iK}\right]$, the corresponding response matrix $A(\Theta_{i})$ is given by

(11) $$A\left(\Theta_{i}\right)=\left[a\left(\vartheta_{i1}\right),a\left(\vartheta_{i2}\right),\ldots ,a\left(\vartheta_{iK}\right)\right]$$

where $A(\Theta_{i})$ is an N×K−dimensional matrix.

Then, the Sto-ML cost function for each group is computed as

(12) $$L\left(\Theta_{i}\right)=\log\left\{det\left[\Pi_{A(\Theta_{i})}R\Pi_{A(\Theta_{i})}+\frac{\mathrm{tr}\left(\Pi_{A(\Theta_{i})}^{⊥}R\right)\Pi_{A(\Theta_{i})}^{⊥}}{N-K}\right]\right\}$$

where det(•) denotes the determinant of the matrix, tr(•) denotes the trace, and ${\left(•\right)}^{-1}$ denotes the matrix inverse. $\Pi_{A(\Theta_{i})}=A\left(\Theta_{i}\right){\left[A^{H}\left(\Theta_{i}\right)A\left(\Theta_{i}\right)\right]}^{-1}A^{H}\left(\Theta_{i}\right)$ is the projection matrix related to the matrix $A(\Theta_{i})$, and its orthogonal complement is given by $\Pi_{A(\Theta_{i})}^{⊥}=I-\Pi_{A(\Theta_{i})}$ with I the N×N-dimensional identity matrix.

The candidate group that minimizes the cost function $L(\Theta_{i})$ is selected, and the *K* angles in that group are taken as the final DOA estimates. The total complexity of the proposed method is $O\left[N^{2}K+G\left(3N^{3}+K^{3}+2NK^{2}+N^{2}K\right)\right]$. And the complete procedure of the proposed DOA estimation algorithm for multi-beam FBS-LWAs is summarized in Algorithm 1.
**Algorithm 1** The steps of proposed method for multi-beam FBS-LWAs.Employ FFT to the signals received by multi-beam FBS-LWAs to obtain X.Calculate the signal covariance matrix R by (6).Obtain the spatial spectrum $P_{CBF}\left(\theta \right)$ through (7) over $\Theta_{FoV}$.Select the first *P* largest peaks of $P_{CBF}\left(\theta \right)$ to form the initial set $\Theta_{\mathrm{ini}}$.Refine $\Theta_{\mathrm{ini}}$ using a finer interval $\theta_{s}=\theta_{g}/2$ by (8) to obtain the candidate set $\Theta_{\mathrm{cand}}$.Generate all K−element combinations from $\Theta_{\mathrm{cand}}$ to form candidates $\Theta_{1},\ldots ,\Theta_{G}$.For each group $\Theta_{i}$, construct the response matrix $A(\Theta_{i})$ by (1) and (11).Calculate the Sto-ML cost function $L(\Theta_{i})$ by (12) for each group.Select the group related to the minimum $L(\Theta_{i})$ as the final DOA estimate.

## 4. Simulation and Performance Analysis

The simulation results of the proposed method are presented in this section, based on the multi-beam FBS-LWA received model. The antenna employed is a single-port multi-beam FBS-LWA with a 50Ω matched load connected to the other port. The design parameters are as follows: the number of operating frequency samples is N=100, with the minimum and maximum operating frequencies set to $f_{\min}=25.5\;\mathrm{GHz}$ and $f_{\max}=27.5\;\mathrm{GHz}$, respectively. The physical length of the antenna is l=20cm, and the harmonic orders radiated at the backward and forward ends are $m_{\mathrm{back}}=-4$ and $m_{\mathrm{forw}}=1$, respectively. Other parameters include a spatial modulation period of p=3.13cm, a relative permittivity of $\varepsilon_{r}=2.94$, a waveguide width of $W_{g}=3.5\;\mathrm{mm}$, and a normalized attenuation constant of $\alpha_{n}/k_{0}\left(n\right)=0.01$.

All simulations were carried out on a Windows 11 system (Microsoft, Redmond, WA, USA) equipped with an Intel i5-10400 CPU (Intel, Santa Clara, CA, USA), using MATLAB R2022b. Unless otherwise specified, the number of snapshots is set to L=100. The FoV of the multi-beam FBS-LWA is $\left[-90^{\circ },90^{\circ }\right]$, with a search grid resolution of $\theta_{g}=2^{\circ }$ and a finer interval of $\theta_{s}=1^{\circ }$ used for angle refinement. To facilitate the plotting of smooth and complete root mean square error (RMSE) curves, the simulation angles in this section are set to the values with decimals.

To comprehensively evaluate the performance of various algorithms for multi-beam FBS-LWAs, the modified enhanced PUMA algorithm with forward-backward spatial smoothing (Mod-EPUMA-FBSS) and the MUSIC algorithm with modified spatial smoothing preprocessing (MUSIC-MSSP), both of which are effective for single-beam FBS-LWAs, are considered for comparison. Note that both methods require transforming the steering matrix into a Vandermonde structure via interpolation. In particular, the MSSP technique uses 10 overlapping subarrays, each containing 90 elements. Meanwhile, since the existing DOA estimation method for coherent signals [26] is designed for a two-port antenna structure, and this paper adopts a single-port configuration, which is not included in the comparison. In addition, the $L_{1}$-norm-based singular value decomposition ($L_{1}$-SVD) method [30], the orthogonal matching pursuit (OMP) algorithm [31], and the sparse iterative covariance-based estimation (SPICE) algorithm [32] are implemented over the search domain $\Theta_{{FoV}^{*}}=-90^{\circ }:\theta_{s}:90^{\circ }$ to illustrate the estimation limitations caused by multi-beam operation. For $L_{1}$-SVD, the regularization hyperparameter is set to 2.

**Figure 3:**
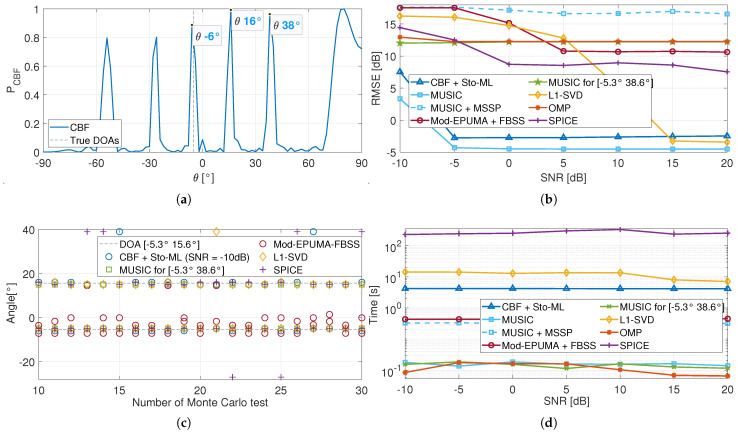
Performance of the proposed method (no description for incoming direction [$-5.3^{\circ }15.6^{\circ }$]). (**a**) CBF spectrum of noise-free signal received by a multi-beam FBS-LWA with incoming directions $-5.3^{\circ }$. (**b**) RMSE performance. (**c**) Partial results in different Monte Carlo tests (no description for SNR = 10 dB). (**d**) Time performance.

Figure 3a presents the spatial spectrum of the noise-free signal received by a multi-beam FBS-LWA with incoming directions $-5.3^{\circ }$, which is obtained by the CBF algorithm. It can be seen that the CBF spectrum exhibits multiple peaks, including the nearest grid point at $-6^{\circ }$, related to the true DOA, and other parasitic peaks. The angles corresponding to the latter are named as the parasitic directions of $-5.3^{\circ }$, which are received at the same operating frequency.

Figure 3b presents the RMSE performance of different methods based on the multi-beam FBS-LWA received model, with incoming directions [$-5.3^{\circ }15.6^{\circ }$], where the DOA of each source coincides with a parasitic direction of the other sources. At low-SNR regions, the sample covariance matrix cannot reflect the signal information under the influence of noise; therefore, the proposed method cannot effectively suppress the interference from the parasitic directions, as shown in Figure 3c, and thus has a high RMSE performance. As the SNR increases, the signal components become more prominent, allowing the proposed method to better exploit the signal structure by maximizing the likelihood function. Therefore, the estimation performance of the proposed algorithm gradually improves and achieves accurate on-grid estimation at SNR = −5 dB, as shown in Figure 3b. Meanwhile, the proposed method exhibits the longest average running time of 4.2 s per experiment, primarily due to the repeated calculation of the cost function, as shown in Figure 3d.

It is worth noting that the MUSIC algorithm appears to achieve accurate on-grid DOA estimation when SNR = −5 dB, despite the full coherence of the sources in this simulation. This phenomenon occurs because the antenna exhibits a slightly stronger response at $15.6^{\circ }$ than at other parasitic angles, as shown in Figure 3a. When the incoming directions are [$-5.3^{\circ }38.6^{\circ }$] and the grid resolution is $2^{\circ }$, the estimation results of MUSIC remain at $-6^{\circ }$ and $16^{\circ }$, as illustrated in Figure 3c, leading to its higher RMSE. Therefore, the MUSIC algorithm is ineffective at suppressing parasitic interference in the multi-beam FBS-LWA received model.

In addition, the interpolation-based methods, such as MUSIC-MSSP and Mod-EPUMA-FBSS, are also negatively affected. The initial angle estimates obtained by the CBF algorithm often fall within parasitic directions, causing a mis-constructed interpolation matrix and thus the estimates around parasitic angles rather than the true DOAs, as presented in Figure 3c. Therefore, the interpolation-based methods are also unsuitable for the multi-beam FBS-LWA scenario and are excluded from further analysis.

Figure 3b also presents the RMSE performance of $L_{1}$-SVD, OMP, and SPICE algorithms. As shown in Figure 3c, the OMP and SPICE algorithms can only successfully estimate the DOA of the received signal in partial Monte Carlo tests, resulting in their high RMSE at SNR = 10 dB. In addition, as the SNR increases, the signal component becomes increasingly dominant over both the parasitic and noise terms. Benefiting from this, the $L_{1}$-SVD algorithm progressively converges to the true DOAs and achieves near on-grid estimation performance when SNR = 15 dB. This high SNR requirement highlights the limited capability of the $L_{1}$-SVD algorithm in suppressing parasitic interference.

**Figure 4:**
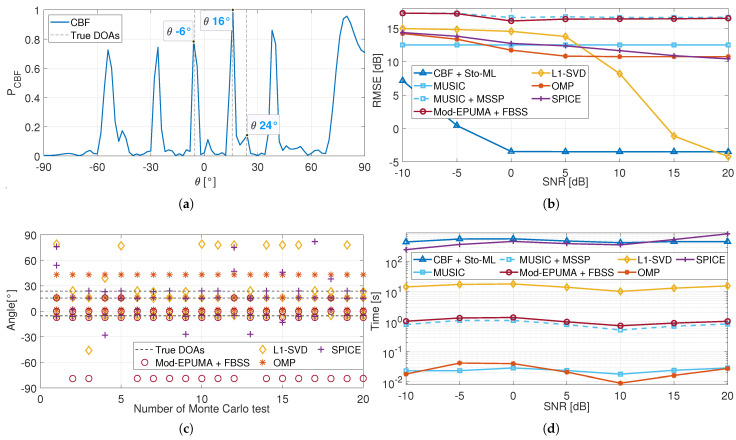
Performance of the proposed method with incoming direction [$-5.3^{\circ }15.6^{\circ }23.7^{\circ }$]. (**a**) CBF spectrum of noise-free signal. (**b**) RMSE performance. (**c**) Partial results in different Monte Carlo tests (SNR = 5 dB). (**d**) Time performance.

Figure 4a shows the CBF spectrum of the noise-free signal received by a multi-beam FBS-LWA, with incoming directions [$-5.3^{\circ }15.6^{\circ }23.7^{\circ }$]. Among these, $-5.3^{\circ }$ and $15.6^{\circ }$ are received simultaneously when the antenna operates at approximately 26.8535 GHz, while $23.7^{\circ }$ is received when the antenna operates at around 25.702 GHz. Due to the superposition effect, the neighboring grids at $-6^{\circ }$ and $16^{\circ }$, corresponding to the true angles $-5.3^{\circ }$ and $15.6^{\circ }$, exhibit more prominent peaks than the neighboring grid at $24^{\circ }$, which corresponds to $23.7^{\circ }$.

Figure 4b compares the RMSE performance of different methods at the aforementioned incoming direction. Due to its limited capability in suppressing parasitic interference, the proposed method exhibits a relatively high RMSE in low-SNR regions. However, as the SNR increases, its estimation performance gradually improves. In this simulation, the number of parasitic angles is greater than in the previous simulation, resulting in stronger parasitic interference. Consequently, the proposed method requires higher SNR conditions to achieve accurate on-grid estimation, which is attained at an SNR of 0 dB. Meanwhile, when the number of sources increases to K=3, the total number of subgroups rises significantly. As a result, the computational burden of repeatedly evaluating the cost function leads to the proposed method exhibiting the longest running time-approximately 487.36 s-as shown in Figure 4d. Future research could focus on reducing the number of subgroups *G* or developing a more efficient cost function to improve estimation efficiency.

Moreover, owing to the rank deficiency of the sample covariance matrix and the challenge posed by the multi-beam operation, the RMSE of the MUSIC algorithms exceeds 10 dB in all SNR regions. Similar to the previous simulation, due to the incorrectly constructed interpolation matrix, the estimates of MUSIC-MSSP and Mod-EPUMA-FBSS are closer to the parasitic directions than to the true DOAs, which leads to their RMSE performance degradation.

As shown in Figure 4c, the OMP and SPICE algorithms can only achieve the true DOAs for partial sources in several Monte Carlo tests. Due to the limited ability of parasitic interference suppression, OMP and SPICE show high RMSE performance in all SNR regions in Figure 4b. In contrast, as the SNR increases, the received signal contains more informative content, which facilitates the convergence of the $L_{1}$-SVD algorithm toward the true DOAs. Thus, the RMSE of the $L_{1}$-SVD algorithm gradually decreases with the SNR increasing and then achieves high-accuracy estimation performance at SNR=20dB. Furthermore, The $L_{1}$-SVD algorithm achieves DOA estimation by solving a second-order cone optimization problem (SCOP), which takes significantly less running time than the Sto-ML algorithm, as shown in Figure 4d. However, it exhibits high SNR requirements and is therefore unsuitable for most practical communication scenarios.

**Figure 5:**
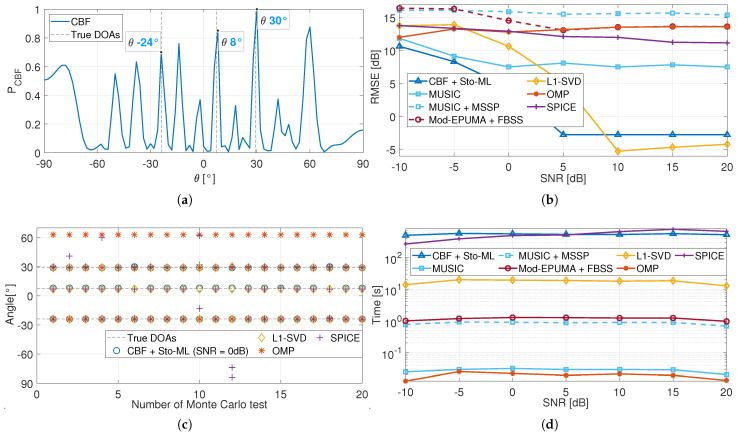
Performance of the proposed method with incoming direction [$-23.8^{\circ }7.2^{\circ }29.4^{\circ }$]. (**a**) CBF spectrum of noise-free signal. (**b**) RMSE performance. (**c**) Partial results in different Monte Carlo tests (no description for SNR = 5 dB). (**d**) Time performance.

Figure 5a shows the CBF spectrum of the noise-free signal received by a multi-beam FBS-LWA with incoming directions [$-23.8^{\circ }7.2^{\circ }29.4^{\circ }$]. Among these, $7.2^{\circ }$ and $29.4^{\circ }$ are received simultaneously when the antenna operates at approximately 26.0859 GHz. In addition, to ensure continuous beam scanning, the antenna must be designed such that the beam pointing angle associated with the *m*th-order spatial harmonic at $f_{max}$ is greater than that associated with the (m+1)th-order harmonic at $f_{min}$. Therefore, $-23.8^{\circ }$ is received when the antenna operates at around 27.096 GHz and 25.5404 GHz. Under this configuration, the sources have similar amplitude in the CBF spectrum, and the total number of parasitic angles is greater than that in Figure 4a.

Figure 5b shows the RMSE performance of different methods with incoming directions [$-23.8^{\circ }7.2^{\circ }29.4^{\circ }$]. Due to the greater number of parasitic directions in this scenario, the set of candidate angles $\Theta_{cand}$ obtained from the initial estimation process may not fully contain the true DOAs. Therefore, the RMSE of the proposed method is about 5 dB when SNR = 0 dB and then becomes flat when SNR = 5 dB. Similarly, the proposed algorithm involves a large number of candidate subgroups due to the larger *K* in this simulation. The repeated evaluations of the cost function result in a longer running time, averaging approximately 541.88 s, as shown in Figure 5d.

In addition, the $L_{1}$-SVD algorithm achieves accurate DOA estimation only at SNR = 10 dB in this scenario. However, when compared with the RMSE curve in Figure 5b, it is indicated that the estimation performance of $L_{1}$-SVD varies significantly with different incoming directions. Therefore, the $L_{1}$-SVD algorithm is also unsuitable for DOA estimation in multi-beam FBS-LWA scenarios. In contrast, the proposed method can effectively suppress the parasitic interference induced by the multi-beam operation and delivers consistent estimation performance in different simulations, demonstrating its strong robustness.

In conclusion, the proposed method can effectively suppress the parasitic interference introduced by the multi-beam operation and successfully achieve accurate DOA estimation in low- and medium-SNR regions, offering a feasible solution to DOA estimation based on the multi-beam FBS-LWA received model. However, due to multiple calculations of the cost function, the total computational cost of the proposed method is high. Therefore, future research may focus on reducing the number of candidate subgroups or exploring more efficient estimation methods based on maximizing the likelihood function.

In contrast, classical subspace-based methods such as MUSIC and Mod-EPUMA are inapplicable under coherent conditions due to covariance matrix rank deficiency. Although interpolation techniques can extend these methods to coherent scenarios, they depend heavily on accurate initial estimates, which are unavailable in multi-beam FBS-LWAs due to parasitic directions. Additionally, parts of sparse reconstruction algorithms, such as $L_{1}$-SVD, OMP, and SPICE, exhibit poor parasitic interference suppression and therefore are also not suitable for application in multi-beam FBS-LWAs.

## 5. Conclusions

To mitigate the parasitic interference introduced by multi-beam operations, this paper proposes a novel DOA estimation method for coherent signals received by multi-beam FBS-LWAs. The proposed method first employs the CBF algorithm for initial estimation, effectively reducing the number of subsequent candidate groups. A grid refinement strategy is then applied to enhance estimation accuracy. Subsequently, the Sto-ML algorithm is utilized to suppress parasitic interference inherent to the multi-beam FBS-LWA received model, thereby achieving accurate DOA estimation. Simulation results under various configurations demonstrate that the proposed method effectively suppresses parasitic angular interference and performs robust and accurate DOA estimation for coherent signals in low-SNR scenarios. Future research may focus on reducing the number of candidate subgroups or exploring more efficient estimation methods based on maximizing the likelihood function.

## Data Availability

Data is contained within the article.
